# Machine learning-based classification of mitochondrial morphology in primary neurons and brain

**DOI:** 10.1038/s41598-021-84528-8

**Published:** 2021-03-04

**Authors:** Garrett M. Fogo, Anthony R. Anzell, Kathleen J. Maheras, Sarita Raghunayakula, Joseph M. Wider, Katlynn J. Emaus, Timothy D. Bryson, Melissa J. Bukowski, Robert W. Neumar, Karin Przyklenk, Thomas H. Sanderson

**Affiliations:** 1 Department of Emergency Medicine, University of Michigan Medical School, Ann Arbor, MI 48109 USA; 2 Neuroscience Graduate Program, University of Michigan Medical School, Ann Arbor, MI 48109 USA; 3 Department of Physiology, Wayne State University School of Medicine, Detroit, MI 48201 USA; 4 Department of Human Genetics, University of Pittsburgh, Pittsburgh, PA 15269 USA; 5 Frankel Cardiovascular Center, University of Michigan Medical School, Ann Arbor, MI 48109 USA; 6 Department of Molecular and Integrative Physiology, University of Michigan Medical School, Ann Arbor, MI 48109 USA

**Keywords:** Neurological disorders, Computational science, Cell death in the nervous system, Molecular neuroscience, Stroke

## Abstract

The mitochondrial network continually undergoes events of fission and fusion. Under physiologic conditions, the network is in equilibrium and is characterized by the presence of both elongated and punctate mitochondria. However, this balanced, homeostatic mitochondrial profile can change morphologic distribution in response to various stressors. Therefore, it is imperative to develop a method that robustly measures mitochondrial morphology with high accuracy. Here, we developed a semi-automated image analysis pipeline for the quantitation of mitochondrial morphology for both in vitro and in vivo applications. The image analysis pipeline was generated and validated utilizing images of primary cortical neurons from transgenic mice, allowing genetic ablation of key components of mitochondrial dynamics. This analysis pipeline was further extended to evaluate mitochondrial morphology in vivo through immunolabeling of brain sections as well as serial block-face scanning electron microscopy. These data demonstrate a highly specific and sensitive method that accurately classifies distinct physiological and pathological mitochondrial morphologies. Furthermore, this workflow employs the use of readily available, free open-source software designed for high throughput image processing, segmentation, and analysis that is customizable to various biological models.

## Introduction

Mitochondria are highly dynamic organelles that exist in a network that is constantly elongating and dividing. Under normal conditions the mitochondrial network is in an equilibrium state of long, interconnected networks and short punctate mitochondria, and maintaining this balanced state is critical for mitochondrial homeostasis, cell stability, and cell survival^1–3^.

Mitochondrial fission (division) and fusion (elongation) events give rise to distinct morphologies. The ever-changing state of mitochondrial morphology is regulated by a family of dynamin-like GTPases, which consist of the fission protein dynamin-related protein 1 (Drp1) as well as the fusion proteins mitofusin (Mfn) 1&2 and optic atrophy 1 (Opa1). Fission of mitochondria plays an important role in segregating unhealthy mitochondria that contain dysfunctional proteins, destabilized membranes, and mutated or damaged mitochondrial DNA (mtDNA)^4–8^. Alternatively, fusion aids in the equilibration of matrix metabolites, intact mtDNA, and membrane components such as electron transport complexes^4,9–11^.

Dramatic alterations in mitochondrial network architecture have been observed in cell cycle progression, cellular differentiation, oxidative stress, metabolic perturbation, and induction of programmed cell death pathways^1,12–18^. Despite the effort to study the phenotypic heterogeneity of mitochondria under these various conditions, investigations have been limited by their measures of mitochondrial morphology, often resorting to manual classification of simple morphologic states, e.g. fused versus fragmented. Studies typically utilize a qualitative or semi-quantitative approach by developing a scoring system of fission/fusion profiles or binning mitochondria based on length^19–22^, both of which lack, on a large-scale, sampling size, and an accurate and precise assessment of physiologically relevant mitochondrial morphologies. To overcome this, recent studies have shifted to utilizing computational image analysis, commonly referred to as image cytometry, which limits observer and selection bias in morphological evaluations and demonstrates high throughput capabilities^23–26^.

Computational image analysis of mitochondrial morphology using machine learning techniques offers the advantages of generating accurate classification and quantitation of different morphologies with fast and efficient large-scale application. Although this method provides robust and accurate analysis of mitochondrial morphology, the availability and expertise required to utilize machine learning software presents a barrier for investigators. Here, we developed a semi-automated image analysis pipeline, that utilizes open-source software for the quantitation of mitochondrial morphology for both in vitro and in vivo applications. Utilizing mouse primary cortical neurons, our pipeline demonstrates a highly specific and sensitive method of efficiently classifying mitochondrial morphology that can be extended to 3D in vivo applications through immuno-labeling of brain sections as well as serial block-face scanning electron microscopy (SBF-SEM) data.

## Results

### Identification of distinct mitochondrial morphologies

**Figure 1:**
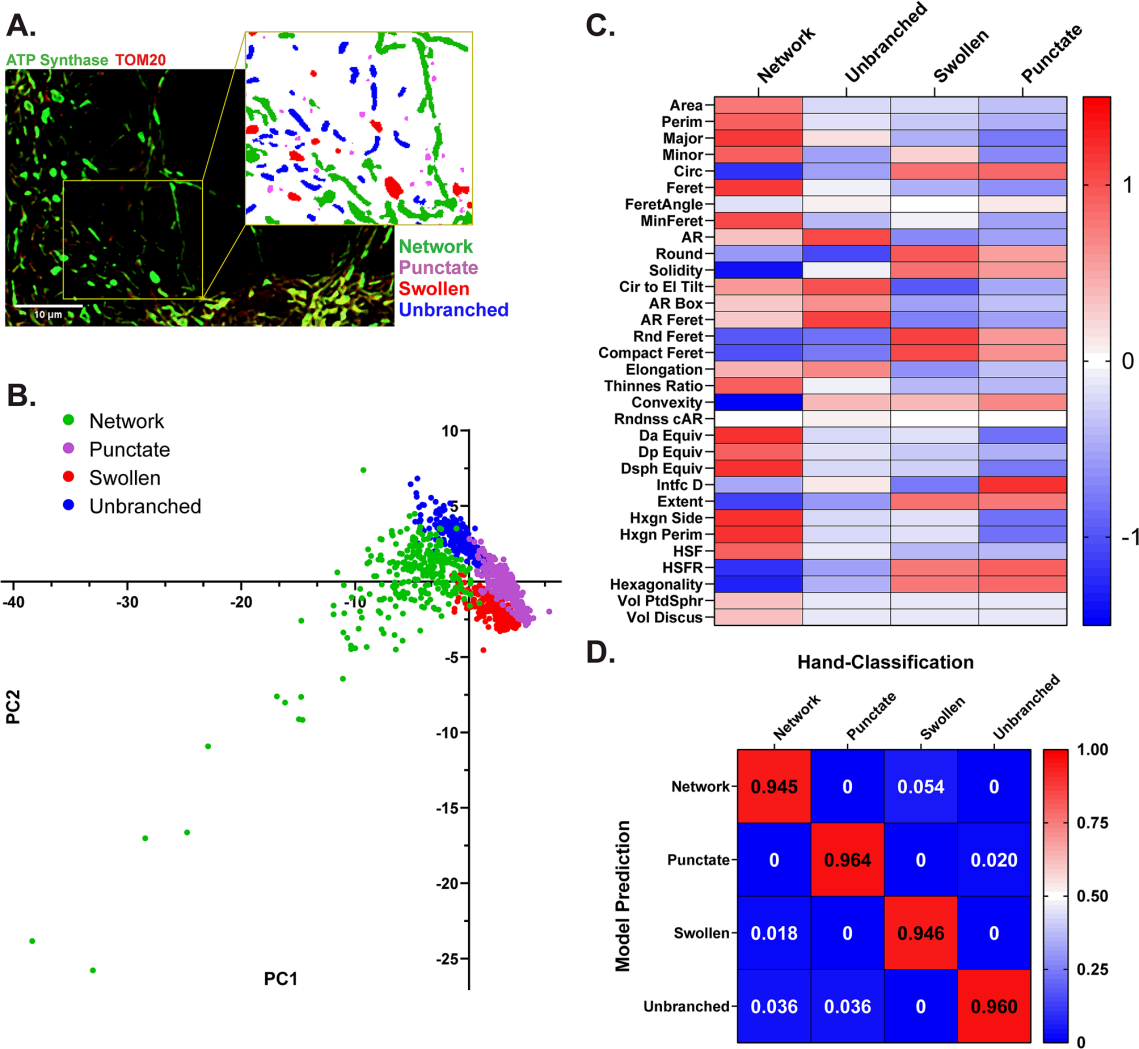
Identification of distinct mitochondrial morphologies in primary neurons. (**A**) Representative immunofluorescent image of mitochondria labeled for ATP synthase (green) and TOM20 (red). Insert: mitochondrial objects segmented and color coded by classified morphology. (**B**) Principal component analysis (PCA) of hand-classified mitochondrial objects in the training and test sets combined. Individual objects are color coded by morphology (n = 1091 mitochondrial objects). (**C**) Heatmap displaying the scaled 32 size and shape measurements by morphology. (**D**) Confusion matrix displaying the results of model evaluation using the hand-classified test set (217 objects).

Mouse primary cortical neurons were cultured on glass coverslips and immuno-labeled with antibodies targeting ATP synthase on the inner mitochondrial membrane and TOM20 on the outer mitochondrial membrane. Fluorescent microscopy revealed four distinct mitochondrial morphologies: network, unbranched, swollen, and punctate that represent frequent mitochondrial size and shape parameters in neurons^19,27^ (Fig. 1A). Networks were defined as long sprawling mitochondrial objects (~ ≥ 5 µm^2^) with one or more branching points. Mitochondria were considered unbranched if they were of intermediate size (~ 1–4 µm^2^), uniform thickness, and did not include a branching point. Punctate objects were defined as small compact objects (~ ≤ 1 µm^2^). Swollen mitochondria were identified as intermediate sized objects (~ 1–4 µm^2^) with high circularity and roundness.

To validate the independence of these observed morphologies, ATP synthase and TOM20 signals were merged, processed together, and mitochondrial objects were segmented and analyzed using a semi-automated image processing workflow in FIJI^28^ (Supplementary Fig. 1). Each mitochondrial object was classified using 32 size and shape descriptors and a principal component analysis (PCA) was applied to evaluate phenotypic differences (Fig. 1B). Mitochondrial objects aggregated at discrete quadrants of the PCA plot (Fig. 1B), further validating that mitochondrial segregate into distinct morphological phenotypes. Comparisons across individual variables, namely roundness (Round) and interfacial density (Intfc D), also demonstrated the independence of each morphology (Fig. 1C).

### Machine learning prediction of morphology

Mitochondrial objects (1091 total) were segmented, measured, and hand-classified for the development of a morphological classification model. This hand-classified data set was split into the machine learning train and test sets (80% and 20%, respectively). The training set was used to develop a random forest classification model using the R caret package^29,30^. In this model, a mitochondrial object is classified through 500 parallel decision trees, in which each tree provides an output vote. Each object is assigned a predicted morphology at the conclusion of all decision trees based on the morphology with the majority of votes. The model accuracy over 25 training repetitions was 94.6%. For verification of model accuracy, the test set of mitochondrial objects was run through the completed decision tree model. Test accuracy was 95.4% on the 217 hand-classified objects in the test set (Fig. 1D). The R functions implemented for model training and evaluation are available on a GitHub repository (https://github.com/sanderson-lab/mitomorphology).

### Physiological relevance of morphological phenotypes

To assess physiological relevance, the in vitro machine learning classification workflow was applied to microscopy imaging data of primary neurons after genetic and pharmacological disruption of mechanisms critical for regulating mitochondrial dynamics. The morphologic state of mitochondria in each image is quantified as the percentage of mitochondrial area classified into each morphological phenotype.

**Figure 2:**
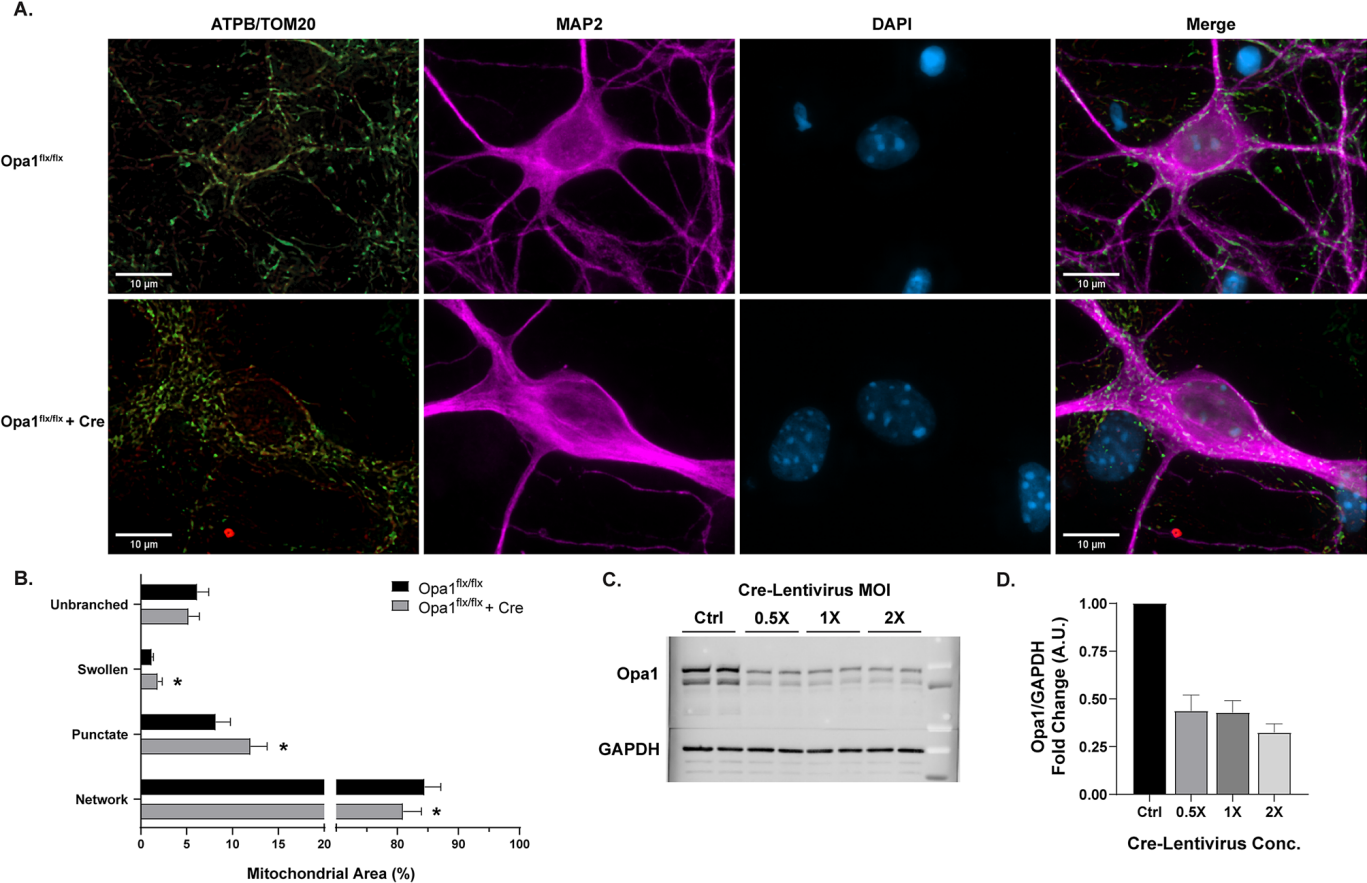
Morphological analysis of mitochondria in Opa1 conditional knockout. (**A**) Representative images of primary *Opa1*^*flx/flx*^ neurons (top) and *Opa1*^*flx/flx*^ neurons with Cre (bottom). Panels (from left to right): ATP synthase (green) and TOM20 (red) merged, MAP2 (magenta), DAPI (blue), all channels merged. Scale bars = 10 µm. (**B**) Percentage of mitochondrial area per morphology in *Opa1*^*flx/flx*^ cells (black) and *Opa1*^*flx/flx*^ cells with Cre (gray). Mean ± SD (n = 9 per group). * indicates *p* < .05. (**C**) Representative Western blot displaying Opa1 and GAPDH protein levels across Cre-Lentivirus concentrations. Full-length blots are presented in Supplementary Fig. 3. (**D**) Western blot quantification of Opa1 protein levels after Cre-Lentivirus treatment (n = 3). For all Opa1 cKO experiments, 1 × MOI Cre-Lentivirus was used.

The dynamin-like GTPase Opa1 is critical for the remodeling of mitochondrial architecture, namely for its role in mitochondrial inner membrane fusion^4,18,31,32^. Disruption of Opa1 prevents mitochondrial fusion, and, based on previous studies using qualitative or semi-quantitative analyses^19–22^, this would be predicted to shift the mitochondrial network toward mitochondrial fragmentation. Primary neurons with conditional knockout (cKO) of Opa1 (Fig. 2A) were generated using primary cells from *Opa1* floxed mice exposed to either lentiviral-Cre (LV- EF1ɑ -Cre) or an empty vector lentivirus (LV- EF1ɑ -empty) as a control (Fig. 2C,D). Opa1 cKO produced a fragmented state, with a decrease in mitochondrial networked area (*p* = .0175) and increases in swollen (*p* = .0015) and punctate area (*p* = .0002; Fig. 2B). These changes in morphology are indicative of impaired mitochondrial fusion and increased cellular stress^15,18,19,32,33^. These results suggest the formation of mitochondrial networks is at least partially dependent on Opa1-mediated fusion, as many others have shown^32–35^.

**Figure 3:**
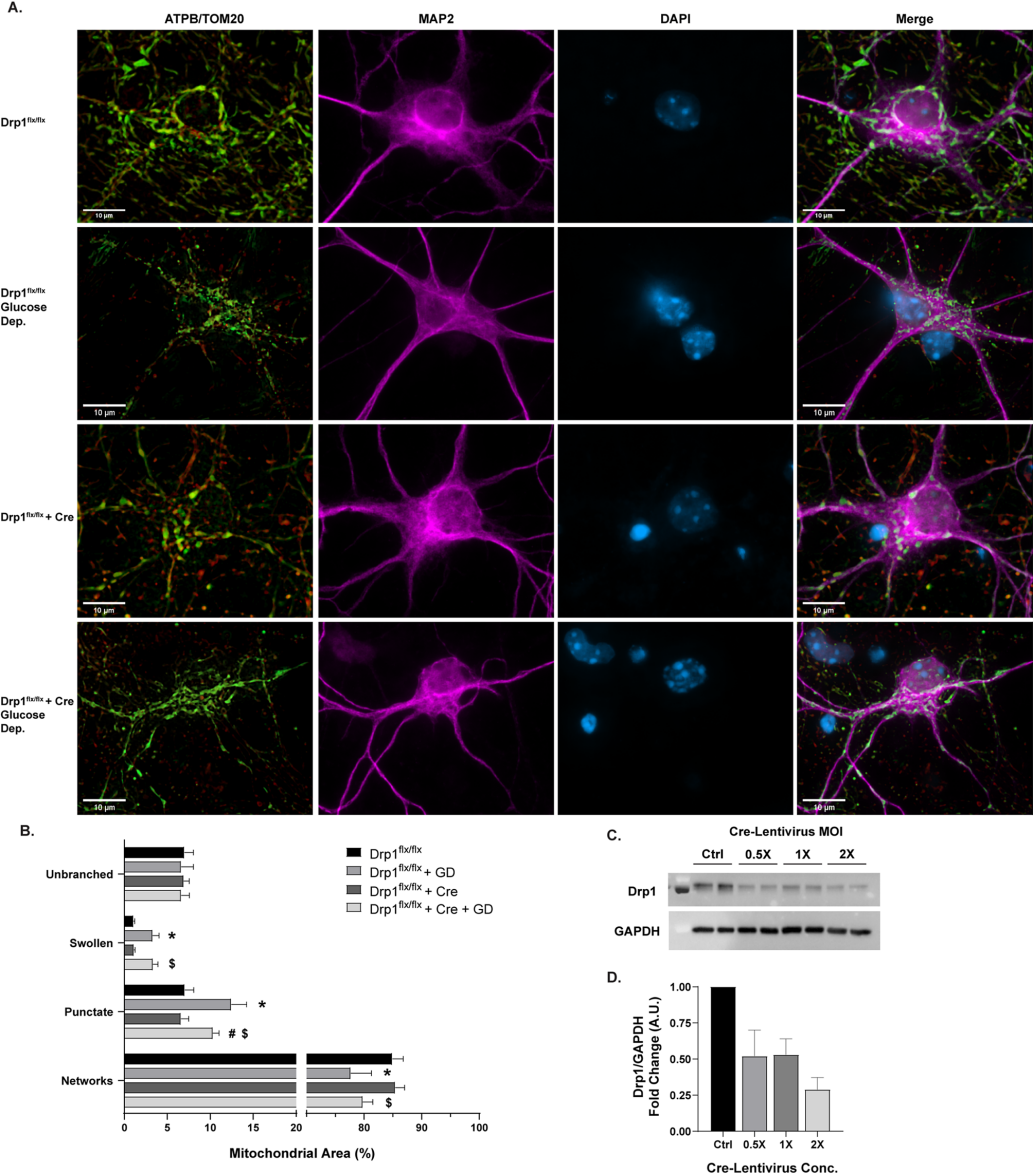
Morphological analysis of mitochondria in Drp1 conditional knockout (cKO) and glucose deprivation (GD). (**A**) Representative images of primary *Drp1*^*flx/flx*^ neurons. Rows (top to bottom): control *Drp1*^*flx/flx*^*, Drp1*^*flx/flx*^ after 18 hr GD, *Drp1*^*flx/flx*^ with Cre, *Drp1*^*flx/flx*^ with Cre after 18 hr GD. Untreated control groups are shown on rows 1 and 3, 18 h GD groups are shown on rows 2 and 4. Panels (from left to right): ATP synthase (green) and TOM20 (red) merged, MAP2 (magenta), DAPI (blue), all channels merged. Scale bars = 10 µm. (**B**) Percentage of mitochondrial area per morphology in Drp1 cKO and GD experiments. Mean ± SD (n = 5–8 per group). * indicates *p* < .05 versus *Drp1*^*flx/flx*^*.* # indicates *p* < .05 versus Drp1^flx/flx^ + GD. $ indicates *p* < .05 versus Drp1^flx/flx^ + Cre. (**C**) Representative Western blot displaying Opa1 and GAPDH protein levels across Cre-Lentivirus concentrations. Full-length blots are presented in Supplementary Fig. 3. (**D**) Western blot quantification of Drp1 protein levels after Cre-Lentivirus treatment (n = 3–4). For all Drp1 cKO experiments, 2 × MOI Cre-Lentivirus was used.

To produce small punctate mitochondria, the GTPase Drp1 will excise and divide mitochondria^13,36,37^. Drp1 cKO was utilized to disrupt mitochondrial fission and shift the mitochondrial network toward a fused network (Fig. 3C,D). Drp1 cKO alone did not have any significant effects on the distribution of morphological phenotypes without cellular stressors (Fig. 3A)^13,15,18,38^. However, when cultures are subjected to 18 h glucose deprivation (GD) as a stressor (Fig. 3A), wild -type (WT) cells show a significant decrease in mitochondrial networked area (*p* = .0003) with concomitant increases in swollen (*p* < .0001) and punctate area (*p* < .0001) (Fig. 3B). Drp1 cKO partially rescued normal mitochondrial morphology resulting in significantly lower percent area of punctate mitochondria (*p* = .0116; Fig. 3B), demonstrating that the GD-induced punctate phenotype is related to Drp1-mediated mitochondrial fission.

**Figure 4:**
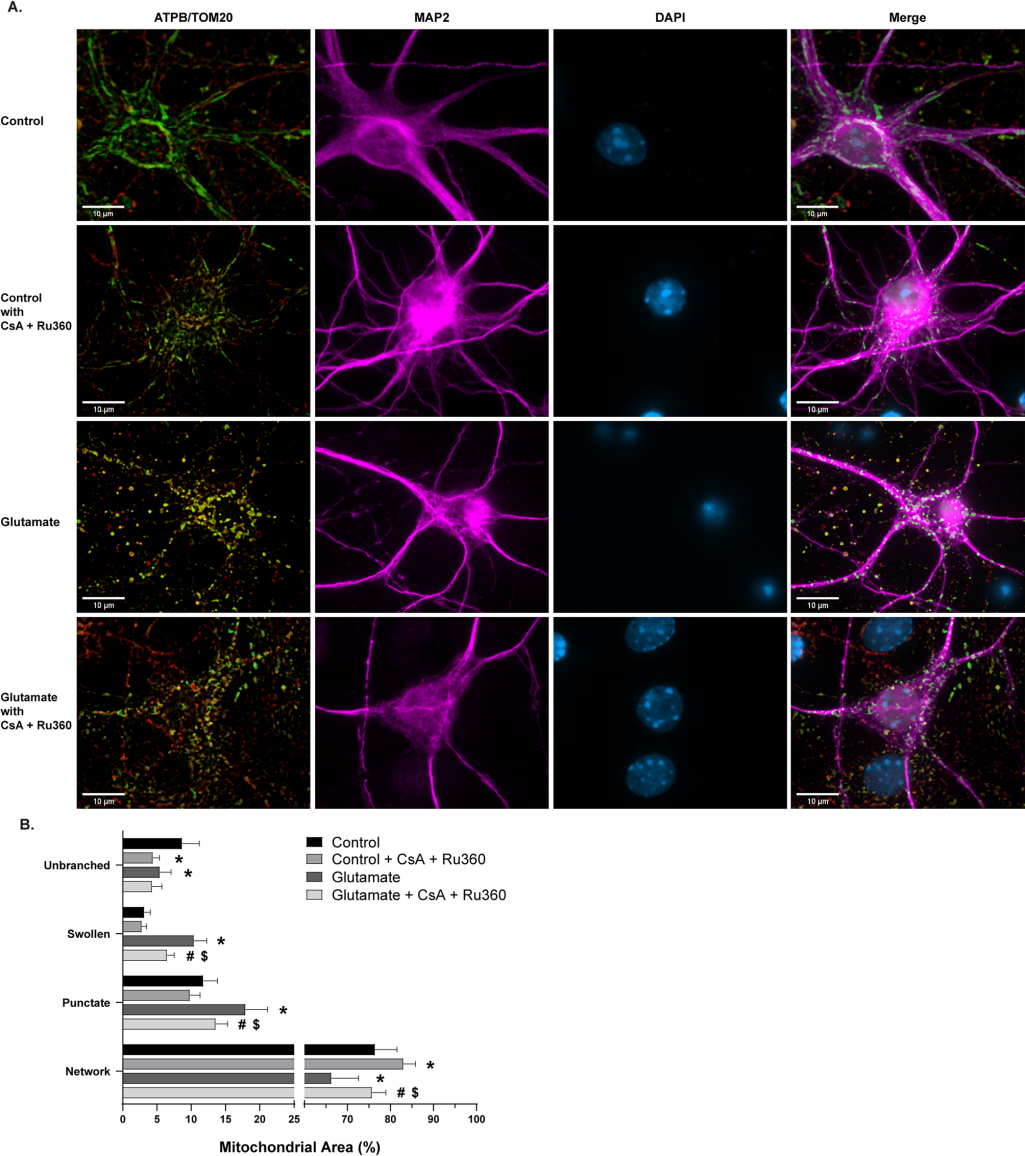
Morphological analysis of mitochondria in glutamate and cyclosporin A (CsA) + Ru360 experiments. (**A**) Representative images of primary cortical neurons. Rows (top to bottom): vehicle-treated control group, control cells treated with CsA + Ru360 only, vehicle-treated group with 30 min 100 µm glutamate exposure, glutamate challenged group pre-treated and co-incubated with CsA + Ru360 Panels (from left to right): ATP synthase (green) and TOM20 (red) merged, MAP2 (magenta), DAPI (blue), all channels merged. Scale bars = 10 µm. (**B**) Percentage of mitochondrial area per morphology in glutamate and CsA + Ru360 experiments. Mean ± SD (n = 6–8 per group). * indicates *p* < .05 versus Control. # indicates *p* < .05 versus Control + CsA + Ru360. $ indicates *p* < .05 versus Glutamate.

A major contributor to mitochondrial dysfunction and the induction of cell death is the formation and activity of the mitochondrial permeability transition pore (mPTP)^15,39–41^. Ca^2+^-induced mitochondrial swelling through the opening of mPTP is a hallmark of cell death in pathological conditions, such as ischemia/reperfusion injury^15,19,41–45^. To assess the reliability of the swollen phenotype and the relevance of this phenotype to pathological mPTP opening and Ca^2+^ influx, cells were exposed to pharmacological modulation of swelling (Fig. 4A). Glutamate and activation of its *N*-Methyl-D-aspartate (NMDA) receptor are known stimulators of mitochondrial swelling and transient mPTP opening^46,47^. Cells treated with 100 µM glutamate and 10 µM co-agonist glycine caused an increase in swollen (*p* < .0001) and punctate mitochondrial area (*p* = .0002), along with a subsequent decrease in networked (*p* = .0025) and unbranched area (*p* = .0118; Fig. 4B). To determine the dependence of this observation on Ca^2+^ handling and mPTP activity, cells were pre-treated and co-incubated with glutamate and the mitochondrial calcium uniporter (MCU) inhibitor Ru360 and mPTP inhibitor cyclosporin A (CsA)^39,48–52^. Treatment with the inhibitors alone decreased unbranched area (*p* = .0004) and increased networked area (*p* = .0449; Fig. 4B), potentially due to the interconnected relationship between Ca^2+^ and Drp1 during remodeling of mitochondria^3,4,53,54^. Furthermore, Ru360 and CsA administration reduced glutamate-induced mitochondrial swelling and fragmentation (Fig. 4B), suggesting the swollen morphological phenotype is associated with mPTP opening and mitochondrial Ca^2+^ homeostasis.

### Workflow application to 3D resolution

**Figure 5:**
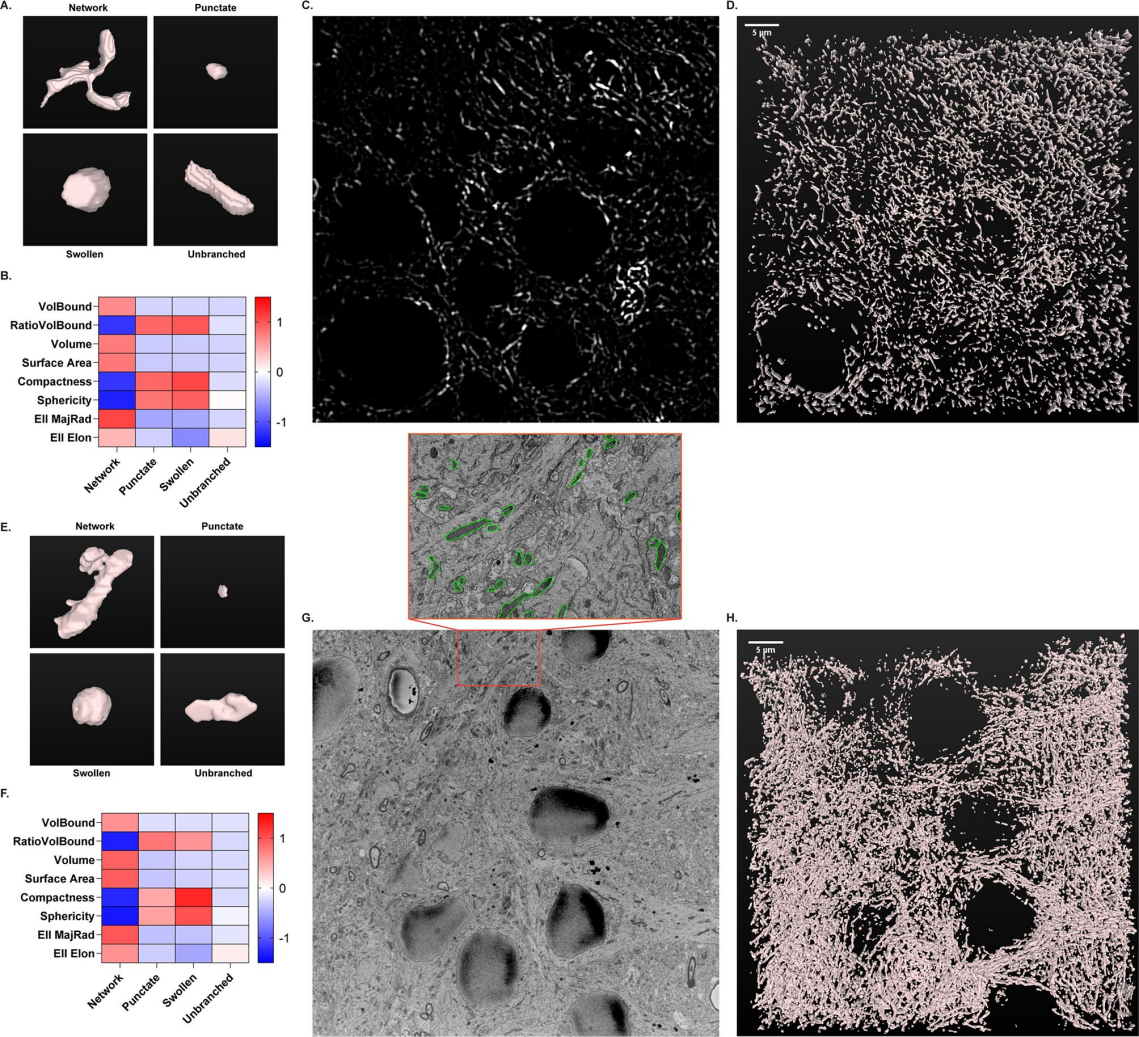
3D morphological classification of mitochondria using confocal (**A**–**C**) and electron microscopy (**D**–**F**). (**A**) Representative 3D renderings of individual mitochondrial objects from mouse brain tissue immuno-labeled for ATP synthase and imaged using confocal microscopy. (**B**) Heatmap displaying the scaled 8 size and shape measurements by morphology for confocal microscopy. (**C**) Representative confocal image of ATP synthase immunofluorescence from mouse hippocampus. (**D**) 3D rendering of mitochondria from the full z-series (50 slices, 5 µm) of the image shown in (**C**), acquired via confocal microscopy. (**E**) Representative 3D renderings of individual mitochondrial objects from rat brain tissue acquired via serial block-face scanning electron microscopy (SBF-SEM). (**F**) Heatmap displaying the scaled 8 size and shape measurements by morphology for SBF-SEM. (**G**) Representative single-plane image from SBF-SEM z-series. Insert: Enlarged area of representative image with mitochondria traced (green). (**H**) 3D rendering of mitochondria from the full z-series (100 slices, 7 µm) of the image shown in (**G**), acquired via SBF-SEM.

A critical limitation of standard morphological analyses of mitochondria, including the in vitro classification model, is the lack of 3D resolution. Cells and organelles have complex three-dimensional architecture and morphology, and inclusion of this 3D resolution can add important details to mitochondrial morphologic analysis. To address this concern, morphological classification workflow was expanded from 2D resolution in cell culture to 3D resolution in tissue histology. Mouse brain tissue sections immuno-labeled for ATP synthase were imaged using fluorescent confocal microscopy of the CA1 hippocampus. Mitochondrial objects were segmented in individual images over a z-series of 50 slices (5 µm total) and stitched together for 3D reconstruction in FIJI using the MorphoLibJ Connected Components Labeling plugin^55^ (Fig. 5A–D). Mitochondrial objects were then measured using 8 size and 3D shape descriptors (Fig. 5B). Through inspection of 3D renderings, mitochondrial objects were hand-classified by morphology (Fig. 5A). Identical to the method described for cell culture, a random forest model was constructed and evaluated using 170 hand-classified mitochondrial objects. Model training with 8 size and 3D descriptors had an accuracy of 76.6%, while the test set was classified with 84.9% accuracy. An expanded descriptor set can be implemented, similar to the primary neuron model, to increase accuracy to meet the needs of each experimental objective.

The same methodology of 3D object measurement, rendering, and classification was applied to serial block-face scanning electron microscopy (SBF-SEM) images of rat brain sections from the CA1 hippocampus (Fig. 5E–H). Mitochondrial objects in individual images were segmented in an automated manner using trainable Weka segmentation^56^ (Supplementary Fig. 2). SBF-SEM sections were analyzed in z-series stacks of 100 slices each (7 µm total). A random forest model was trained and tested on 231 hand-classified mitochondrial objects. The accuracy for training and testing was 74.7% and 86.4%, respectively. Our confocal and SBF-SEM imaging results serve as primary evidence that this analytical pipeline can be scaled to 3D resolution with versatility and reliability.

## Discussion

Mitochondrial morphology is a representative snapshot of the underlying processes of mitochondrial dynamics and quality control. Emerging evidence suggests that the paradigm of mitochondria as effectors of injury extends beyond the integrity of the organelle to encompass the balance between fission and fusion phenotypes and the interface with processes responsible for cell death. Analyses of these morphologic alterations have traditionally been performed using qualitative approaches or simple quantitative measurements^19–22^. However, recent advances in imaging techniques and the availability of computational resources make more advanced analysis of morphology feasible. Several groups have developed software and codes/macros for the unbiased measurement and classification of mitochondrial morphology^23–26^. To further these efforts, we developed a machine-learning based classification pipeline for the identification of distinct physiological and pathological morphologies using free and open-source software with 2D and 3D capabilities.

Utilizing software and plugins freely and readily available to investigators in the biomedical sciences, we designed a workflow for high throughput image processing, segmentation, and analysis that is customizable to various biological models^28,29,55,56^. Starting in cell culture, we identified morphological phenotypes that we hypothesized to be indicative of physiological and pathological mechanisms of mitochondrial dynamics. Using a combination of size and geometric descriptors, we verified the distinct nature of these phenotypes and our ability to classify them by visual observation. Through the application of machine learning in the R caret package, we developed a classification model with high sensitivity and specificity for these morphological phenotypes^29^. Implementation of machine learning classification and semi-automated image processing removes opportunity for bias and inconsistency in morphological analysis, thereby increasing the rigor and reproducibility of these procedures^23,57^. Furthermore, we have demonstrated the utility and versatility of our pipeline through 3D analysis in confocal and SBF-SEM imaging. The applications of our workflow in 3D serves as proof-of-concept that high throughput analyses of mitochondrial morphology can be accomplished with high sensitivity in larger biological models. This technique has the potential to be a significant advancement to standard morphological analysis of mitochondria in cell culture, intact tissue samples, and tissue in vivo live imaging.

Mitochondrial analyses in cell culture models have traditionally been performed with 2D resolution, as many cell types grow in a monolayer in vitro. Imaging and analyzing in 2D reduces the need for advanced imaging techniques and high computational power. However, cells and their organelles exist in 3D and therefore 2D analyses may not provide precise results. To address this limitation, we utilized our 2D workflow as a foundation for the construction of an adaptable 3D analysis methodology. Additionally, a critical limitation of the analyses performed in this study is the lack of cell type specificity. The mitochondrial labeling strategies implemented were not specific to neurons. It is therefore likely that mitochondria from surrounding glial cells and other cells of neurovascular unit were included. The methodologies we have developed have tremendous utility for future studies with research questions relating to specific cell types. Our workflows are easily adaptable for cell type specific mitochondrial labeling strategies, namely genetically encoded fluorescent reporters targeted to mitochondria.

Although our workflow will require evaluation and confirmation in other cell types, tissues, and disease models, we believe our approach has the potential to be customizable for an investigator’s biological model of interest and research question. This versatility is a great asset, but also presents its own limitations. As is always the case with machine learning, a classification model is only as good as its training data. Development of a reliable and sensitive model is dependent on consistent and accurate hand-classification of the train/test set and useful input variables. Inappropriate selection of input variables or inconsistent hand-classification can lead to faulty models. Additionally, with high throughput image analysis, it is critical to verify objects of interest are not lost or altered in processing and segmentation. As an example, during analysis of 3D mitochondrial objects, we observed a “ballooning” effect in which binary objects after segmentation were enlarged compared to the mitochondrial signal in the original images. This effect was reduced through the implementation of a morphological erosion step during binary image processing^19^. A limitation specific to automated SBF-SEM analysis is segmentation based on pixel intensity. We trained a Weka segmentation model for the automated extraction of mitochondrial objects from SBF-SEM images. This technique greatly improved our sampling ability and analysis efficiency. However, application of this method generated a “survivor”-like bias, where mitochondria with severe cristae dysfunction were unintentionally discarded during segmentation, leading to a likely underestimation of swollen mitochondria. It is important to note that application of our proposed pipeline is also possible without automated segmentation. Therefore, possible biases introduced during automated processing and segmentation can be avoided by implementing the classification protocol without batched image segmentation.

There is an increasing need to bridge the fields of computer science, mathematics, and artificial intelligence with the biomedical sciences. Detailed analysis with computational and imaging techniques will greatly advance our understanding of complex biological processes and increase reproducibility in research. Here we describe a semi-automated machine learning-based workflow for the 2D and 3D analysis of mitochondrial morphology using free and open-source software. Our method of morphological classification offers much needed versatility, such that investigators can apply the technique to their biological model and imaging technique of interest. The application of this quantitative method will greatly increase the efficiency and reproducibility of assessing mitochondrial morphology throughout the field.

## Methods

### Animals

All procedures were performed in accordance with institutional and ARRIVE guidelines and approved by the University of Michigan and Wayne State University Institutional Animal Care and Use Committees (IACUC). Mice were maintained on a 12 h light/dark cycle with standard rodent chow and water available ad libitum. Wild-type (WT) mice (C57BL/6Crl) were purchased from Charles River Laboratories (Wilmington, MA). *Drp1* floxed *(Drp1*^*flx/flx*^) mice (*Dnm1l*^*tm1.1Hise*^) were generously provided by Hiromi Sesaki, Johns Hopkins University, Baltimore, MD^58^. *Opa1* floxed (*Opa1*^*flx/flx*^) mice were generously provided by Luca Scorrano, University of Padua, Italy^59^. All mouse models were backcrossed to C57BL/6Crl background. Sprague Dawley rats were obtained from Envigo (Indianapolis, IN).

### Primary neuron culture

Postnatal day 0–2 (P0–P2) mouse pups were sacrificed by decapitation. Cerebral cortices were dissected and minced prior to incubation in enzyme digestion solution (1 × hibernate complete medium, 0.06 mg/mL l-cysteine (Sigma, 778672), 1.4 × 10^−2^ N NaOH (Sigma, 43617), 10 ng/mL APV (2-Amino-5-phosphonopentanoic acid, Sigma, A-5282), with 50 uL Papain (Worthington, LS 03126)) for 30 min at 37 °C. Following digestion, tissue was washed with DPBS and dissociated in complete Hibernate-A medium. Cells were seeded onto 0.1% PEI-coated glass coverslips at a density of 160,000 cells/cm^2^. After 30 min, complete media change was performed with neurobasal complete medium (1 × Neurobasal Plus medium (Gibco, A3582901), 1% B27 Plus (Gibco, A3653401), 0.5 mM Glutamax Supplement (Gibco, 35050061), and 1 Penicillin/Streptomycin Solution (Gibco, SV30010)). Cells were incubated at 37 °C in 5% CO_2_ for 14 days. Half-media changes were performed every 3–4 days with neurobasal complete medium.

### Lentiviral transduction

Lentiviral infection was performed 7 days prior to experimentation, on day-in-vitro 7 (DIV7). Lentiviral plasmids and lentivirus were generated by the vector core at the University of Michigan: Lenti-EF1ɑ-Cre-VSVG, Lenti-EF1ɑ-VSVG. Viruses were prepared in Neurobasal Medium (Gibco, 21103049). For cKO experiments, an empty vector lentivirus (Lenti-EF1ɑ-VSVG) was administered to floxed cells to serve as controls.

### Pharmacological treatment

For glucose deprivation, cells were incubated in EBSS no glucose medium ((0.20 g/L CaCl_2_ (Spectrum, CA138500GM), 0.4 g/L KCl (Fisher Chemical, P217-500), 0.097 g/L MgSO_4_ (Fisher Chemical, M65-500), 6.8 g/L NaCl (Fisher Chemical, S2711), 2.2 NaHCO_3_ (Acros Organics, AC447102500), 0.14 g/L NaH_2_PO_4_-H_2_O (Fisher Chemical, S369-500), and 0.01 g/L Phenol red (Fisher Chemical, P74-10)) for 18 h at 37 °C. For glutamate treatment, cells were incubated in 100 μM glutamate (Sigma, G8415) and 10 μM glycine (Sigma-Aldrich, G5417) in EBSS for 30 min at 37 °C. Prior to glutamate treatment, cells were pre-treated with 1 μM Cyclosporin-A (CsA, Sigma-Aldrich, SML1018) and 10 μM Ru360 (Calbiochem, 557440) for 60 min. Following pre-treatment, cells were incubated with CsA and Ru360 concurrently with glutamate treatment for 30 min.

### Cryostat sectioning

Mice were anesthetized with isoflurane then transcardially perfused with 10 mL of 1 × PBS, followed by 10 mL of 4% PFA in 1 × PBS. Brains were post-fixed overnight in 4% PFA at 4 °C, then cryoprotected in 25% sucrose in 1 × PBS. Brains were cut into 20 µm sections using a cryostat (CM1860, Leica Microsystems), set at − 22 °C, cold mounted onto slides (Superfrost, Thermo Fisher Scientific), and stored at − 20 °C until staining.

### Western blot

Cells were scraped in homogenization-A buffer, sonicated, and protein concentration was measured using the Bradford Plus Assay Reagent (ThermoFisher, PI23236). Polyacrylamide gels (10% 29:1 polyacrylamide/bisacrylamide (Fisher BioReagents, BP1408-1), 375 mM Tris pH 8.8 (Fisher BioReagents, BP152-1), 0.1% sodium dodecyl sulfate (SDS, Sigma, L3771), 0.1% ammonium persulfate (APS, Sigma, #A3678), and 0.1% TEMED (GE Healthcare, # 45-000-226)) were loaded with 10 μg of whole cell lysates and transferred to nitrocellulose membranes. Membranes were incubated with primary antibodies (1:1000 Mouse Anti-Dlp1/Drp1 in 2% milk (Clone 8, BD Transduction Labroraotries, DB6111112); 1:1000 Mouse Anti-Opa1 in 2% BSA (DB Transduction Laboratories, DB612607); 1:10,000 Rabbit Anti-GAPDH in 2% BSA (14C10, Cell Signaling, 2118)) at 4 °C overnight. Membranes were then washed with Tris-Buffered Saline and 0.1% Tween (TBST, Fisher Scientific, BP337500) and incubated in secondary antibodies for 60 min at room temperature. Membranes were then washed 3 × with TBST for 5 min. The membranes were incubated in SuperSignal West Pico Plus Chemiluminescent Substrate (ThermoFisher, 34577), imaged utilizing a BioRad ChemiDoc XRS and imager, and finally quantified by densitometry using ImageJ.

### Immunofluorescence

#### Cell culture

 Cells were fixed with 4% paraformaldehyde (ThermoFisher, 50980487) for 15 min at 37 °C. Coverslips were incubated in blocking solution (5% goat serum (Sigma, G9023) and 0.3% Triton-X100 (Acros Organics, 215682500) in PBS) for 60 min. Coverslips were then incubated in primary antibody solution (1:1000 Mouse Anti-ATPB (Abcam, ab14730), 1:200 Rabbit Anti-TOM20 (ProteinTech, 11802–1-AP), 1:1000 Chicken Anti-MAP2 (Abcam, ab5392), 1% BSA (Sigma, A9647) and 0.3% Triton-X100 in PBS) at 4 °C overnight. After primary incubation, coverslips were washed 3 × in PBS and incubated in secondary antibody solution (1:200 Anti-Mouse Alexa Fluor 488 (Invitrogen, A11029), 1:200 Anti-Rabbit Alexa Fluor 546 (Invitrogen, A10040), 1:200 Anti-Chicken Alexa Fluor 647 (Invitrogen, A21449), 1% BSA (Sigma, A9647) and 0.3% Triton-X100 in PBS) for 60 min. Following secondary incubation, coverslips were washed 3 × with PBS and mounted on glass slides using Fluoroshield with DAPI (Sigma, F6057).

#### Tissue sections

 Slides were permeabilized with 1% triton in 1 × PBS for 10 min and then 3% peroxide for 10 min. Slides were blocked using 2% goat serum/TBSGBA (Tris buffered saline, gelatin, bovine serum, azide) for 1 h. Primary antibody (1:100 Mouse Anti-ATPB, Abcam, ab14730) in blocking solution was incubated overnight at room temperature. Slides were washed with 1 × PBS, incubated with secondary antibody (1:100 Anti-Mouse Alexa Fluor 488 (Invitrogen, A11029) and DAPI (Sigma) for 3 h at room temperature and mounted with ProLong Glass mountant (Thermo Fisher Scientific).

### Fluorescent microscopy

#### Cell culture

 Each coverslip was imaged using a Zeiss Axio Observer Z1 inverted microscope with LED illumination. For each coverslip, 8–10 image z-stacks (0.24 µm slices) were acquired at 63 × with oil immersion. Z-stacks were then deconvolved using the Zeiss Zen Pro regularized inverse filter method. Following, z-stacks were processed using Zeiss Zen Pro extended depth of focus wavelets method. Images were exported in TIFF format for post-processing.

#### Tissue sections

 Images were acquired on a Leica TCS SP8 DMi8 STED Inverted Confocal microscope (Leica Wetzler, Germany) using Leica Application Suite X (LASX). Z-series of 5 μm were collected beginning at the top of the tissue section inward using the following parameters: 400 Hz, z step size:0.1 um, 2048 × 2048. After acquisition, image stacks were deconvolved with Huygens Professional version 19.04 (Scientific Volume Imaging, The Netherlands, http://svi.nl) using CMLE algorithm, with SNR:20 and 40 iterations. Z stacks were exported in TIFF format for post-processing.

### Electron microscopy

Brains were fixed with 2.5% glutaraldehyde then embedded and stained with osmium tetroxide and imaged on a scanning block face scanning electron microscope^19^. Block-face scans of the sample are taken, followed by removal of a 70 nm section and subsequent SEM image the next z-plane. This process is repeated on average 400 to 500 times, generating z-stacks of SEM data at 70 nm intervals.

### Image processing and segmentation

#### Immunofluorescence

Post-processing was performed in FIJI^28^. Images collected from in vitro experiments were imported as merged images of ATP synthase and TOM20 signal. Z series of mouse cerebral tissue were unstacked and imported into FIJI as individual images of ATP synthase signal. The following steps were performed using FIJI’s batch processing feature. Background noise was removed using a rolling ball radius of 10 pixels. Images were filtered via Unsharp Mask with a radius of 1 pixel and mask weight of 0.60. Enhancement of local contrast was performed using Contrast Limited Adaptive Histogram Equalization (CLAHE)^60^ with 256 histogram bins. A median filter was then applied to each image with a radius of 2 pixels. Mitochondria were segmented using Trainable Weka Segmentation^56^. The segmentation classifier model was trained using hand identified mitochondria from processed images, positive for ATP Synthase and/or TOM20 signal. Segmentation output images were converted to 8-bit binary images and the known scale was set. For the identification of mitochondrial objects in 2D, the FIJI Particle Analyzer plug-in was run with a minimum object size of 0.30 um. Measurements from identified objects were then expanded using the Extended Geometric Descriptions macro^61^.

#### Electron microscopy

 Images were imported into FIJI software and 5 × compressed via bilinear interpolation. Mitochondrial objects were segmented from downsized images using Trainable Weka Segmentation^56^. Output images were converted to 8-bit binary.

### 3D object mapping and measurement

Segmented binary images from tissue immunofluorescence and electron microscopy were morphologically eroded using MorphoLibJ Morphological Filters plug-in^55^. Individual images were then re-assembled into z stacks and 3D objects were identified using the MorphoLibJ Connected Components Labeling plug-in at a connectivity setting of 6^55^. Resulting connected objects were measured with the 3D ROI Manager^62^.

### Machine learning classification

Machine learning-based classification of mitochondrial objects was performed in R computing language using the R Caret package^29,30^. Rstudio software (Boston, MA) was used for all R computation. The following procedures were utilized for the development of all classification models described. The measurements of classified mitochondrial objects, along with their classification, were imported into Rstudio as the train/test set. This data set was then split into the training (80%) and test (20%) sets using the createDataPartition function. A random forest algorithm (“rf”) was trained by the training set with 25 repetitions. The test set was then run through the model for the assessment of accuracy. Confusion matrices and principal component analyses were produced to assess the performance of the models.

After model construction, data from in vitro experiments was imported into Rstudio and run through the trained model using the predict function. Predicted morphological phenotypes for each mitochondrial object were then exported along with the original measurements as a csv file. Data was then compiled and organized in Microsoft Excel (Redmond, WA) to compute the percent mitochondrial area belonging to each morphology in a given image/condition. R functions written for model training and morphological predictions are available in our GitHub repository (https://github.com/sanderson-lab/mitomorphology).

### Statistical analysis

Statistical analyses were performed in GraphPad Prism 8 (GraphPad Software, San Diego, CA). For comparisons of two discrete groups, one-way ANOVAs were performed, followed by post-hoc t-tests. For comparisons of more than two groups, two-way ANOVAs were performed with post-hoc comparisons made by Tukey’s test corrected for multiple comparisons. Tests with *p* < .05 were considered statistically significant. The number of biological replicates is indicated by n, unless otherwise noted.

## Supplementary Information

Supplementary Information.

## Data Availability

R functions written for machine learning model training and morphological predictions, as well as sample raw images, are available in our GitHub repository (https://github.com/sanderson-lab/mitomorphology)^29,30^.
