# Supplementary material for: Machine learning-based classification of mitochondrial morphology in primary neurons and brain
Source: Sci Rep. 2021 Mar 4;11:5133. doi: 10.1038/s41598-021-84528-8 (PMC7933342; doi:10.1038/s41598-021-84528-8)
Supplement: Supplementary file 1 — Supplementary Information. [file 41598_2021_84528_MOESM1_ESM.docx]

**Machine Learning-Based Classification of Mitochondrial Morphology in Primary Neurons and Brain**

Garrett M. Fogo^1,2,#^, Anthony R. Anzell^1,3,4,#^, Kathleen J. Maheras^1^, Sarita Raghunayakula^1^, Joseph M. Wider^1^, Katlynn J. Emaus^1,2^, Timothy D. Bryson^1,5^, Melissa J. Bukowski^3^, Robert W. Neumar^1^, Karin Przyklenk^3^, Thomas H. Sanderson^1,2,5,6,*^

^1^ Department of Emergency Medicine, University of Michigan Medical School, Ann Arbor, MI 48109, USA

^2^ Neuroscience Graduate Program, University of Michigan Medical School, Ann Arbor, MI 48109, USA

^3^ Department of Physiology, Wayne State University School of Medicine, Detroit, MI 48201, USA

^4^ Department of Human Genetics, University of Pittsburgh, Pittsburgh, PA 15269, USA

^5^ Frankel Cardiovascular Center, University of Michigan Medical School, Ann Arbor, MI 48109, USA

^6^ Department of Molecular and Integrative Physiology, University of Michigan Medical School, Ann Arbor, MI 48109, USA

^#^These authors contributed equally to this work

*Correspondence

Thomas H. Sanderson, Department of Emergency Medicine, University of Michigan Medical School, Ann Arbor, MI 48109, USA. E-mail address: thsand@umich.edu

Short title: Machine Learning Mitochondrial Classification

**Supplementary Methods**

**Expanded Image Processing & Segmentation**

The following ImageJ/FIJI ^27^ functions (IJ Macro script shown below each step) were executed in batch for the processing of immunofluorescent images (ATPB and TOM20 merged TIFF exports from microscope software) from primary cortical neurons:

Process -> Subtract Background (Rolling Ball Radius = 10.0 pixels)

run("Subtract Background...", "rolling=10");

Process -> Filters -> Unsharp Mask (Radius = 1.0 pixels, Mask Weight = 0.60)

run("Unsharp Mask...", "radius=1 mask=0.60");

Process -> Enhance Local Contrast CLAHE (Blocksize = 127, Histogram Bins = 256, Maximum Slope = 3.00)

run("Enhance Local Contrast (CLAHE)", "blocksize=127 histogram=256 maximum=3 mask=*None* fast_(less_accurate)");

Process -> Filters -> Median (Radius = 2.0 pixels)

run("Median...", "radius=2");

Run Trainable Weka Segmentation Plugin ^54^ (with trained segmentation model)

Image -> Type -> 8-bit

run("8-bit");

Process -> Binary -> Make Binary

run("Convert to Mask");

Analyze -> Set Scale…

run("Set Scale...", "distance=*** known=*** unit=***");

Analyze -> Analyze Particles… (Size = 0.30-Infinity)

run("Analyze Particles...", "size=0.3-Infinity show=Masks display");

Run the Extended Geometric Descriptions macro^59^

Representative images from each step in the processing workflow are displayed in Supplementary Fig. 1.

**Electron Microscopy Hand Segmentation**

For the assessment of our automated segmentation method in analyzing SBF-SEM images, a random sampling of SBF-SEM images were hand-segmented independently by two experienced researchers blinded to automated segmentation methodology and results. The following was used as the criteria for mitochondria: electron dense objects of relatively tubular or rounded morphology, clear organelle boundaries, and visible striations, invaginations, and/or compartments within the organelle structure. The accuracy of automated Weka segmentation quantified by comparison to hand segmented images, via Jaccard Index and Dice Coefficient. Representative images and results are shown in Supplementary Fig. 2.


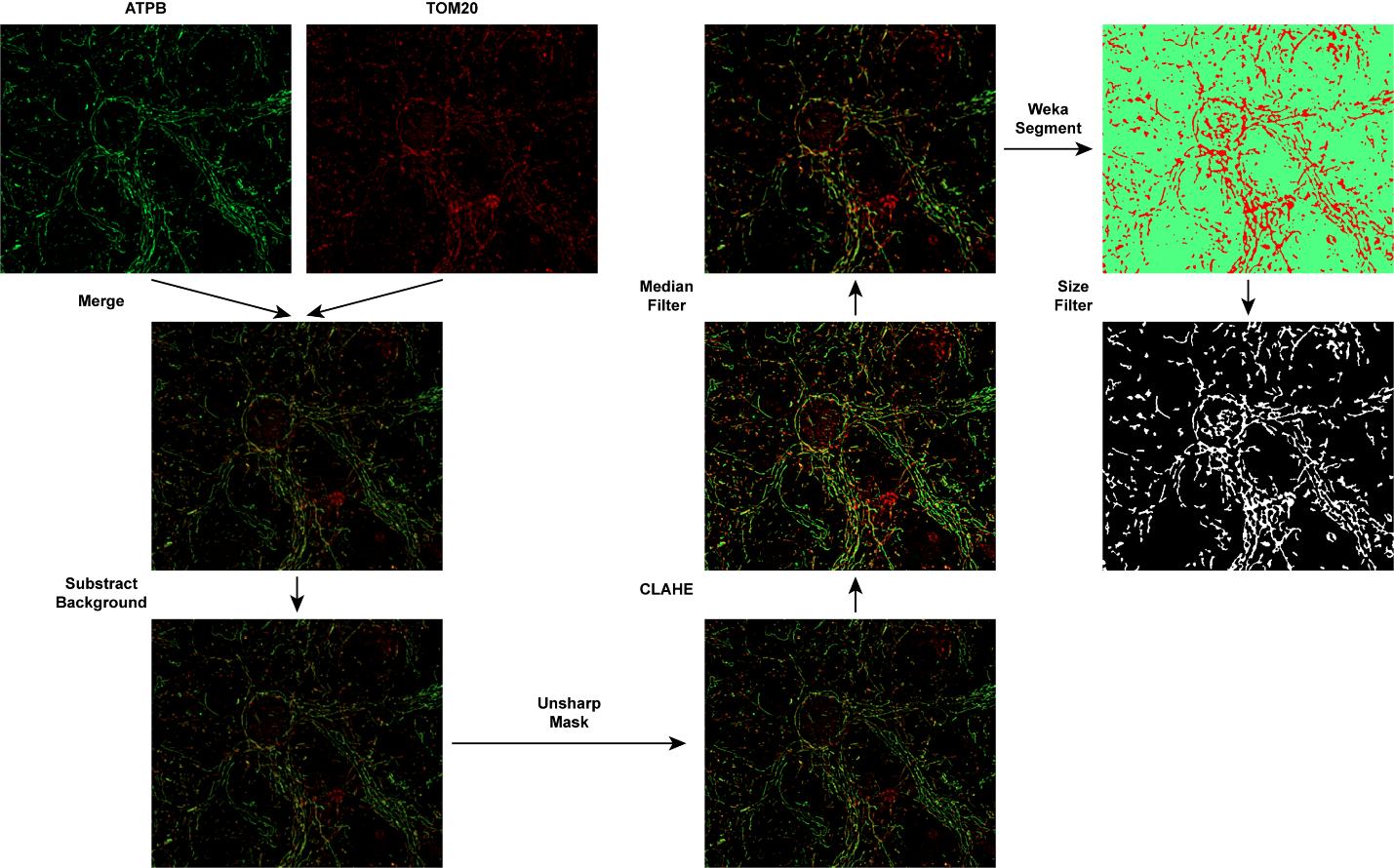


**Supplementary Figure 1. Processing workflow for the segmentation of mitochondrial objects**. Representative ATPB and TOM20 immunofluorescent images were run through the batch processing workflow and images are shown at each processing step. The brightness and contrast of raw ATB and TOM20 images were enhanced for the readability of this figure.





**Supplementary Figure 2. Machine learning-based automated segmentation of SBF-SEM images**. The trainable Weka segmentation plug-in was utilized to segment mitochondria from SBF-SEM images in an automated manner in FIJI ^27,54^. Upper Left: representative raw SBF-SEM image; Upper Right: mitochondrial objects hand segmented by an experienced reviewer; Lower Left: mitochondrial objects segmented via automated Weka segmentation; Lower right: comparison of hand segmented and Weka segmented binary object maps, false positive signal is shown in blue, false negative signal is shown in red. Weka segmentation was performed using a random forest machine learning model (100 decision tree, 8 random features per tree) with a training error of 0.525%. Similarity between hand segmented and Weka segmented object maps was quantified by Jaccard Index (0.575 ± 0.067) and Dice Coefficient (0.7277 ± 0.055) for a set of randomly selected SBF-SEM images (n=9).

**
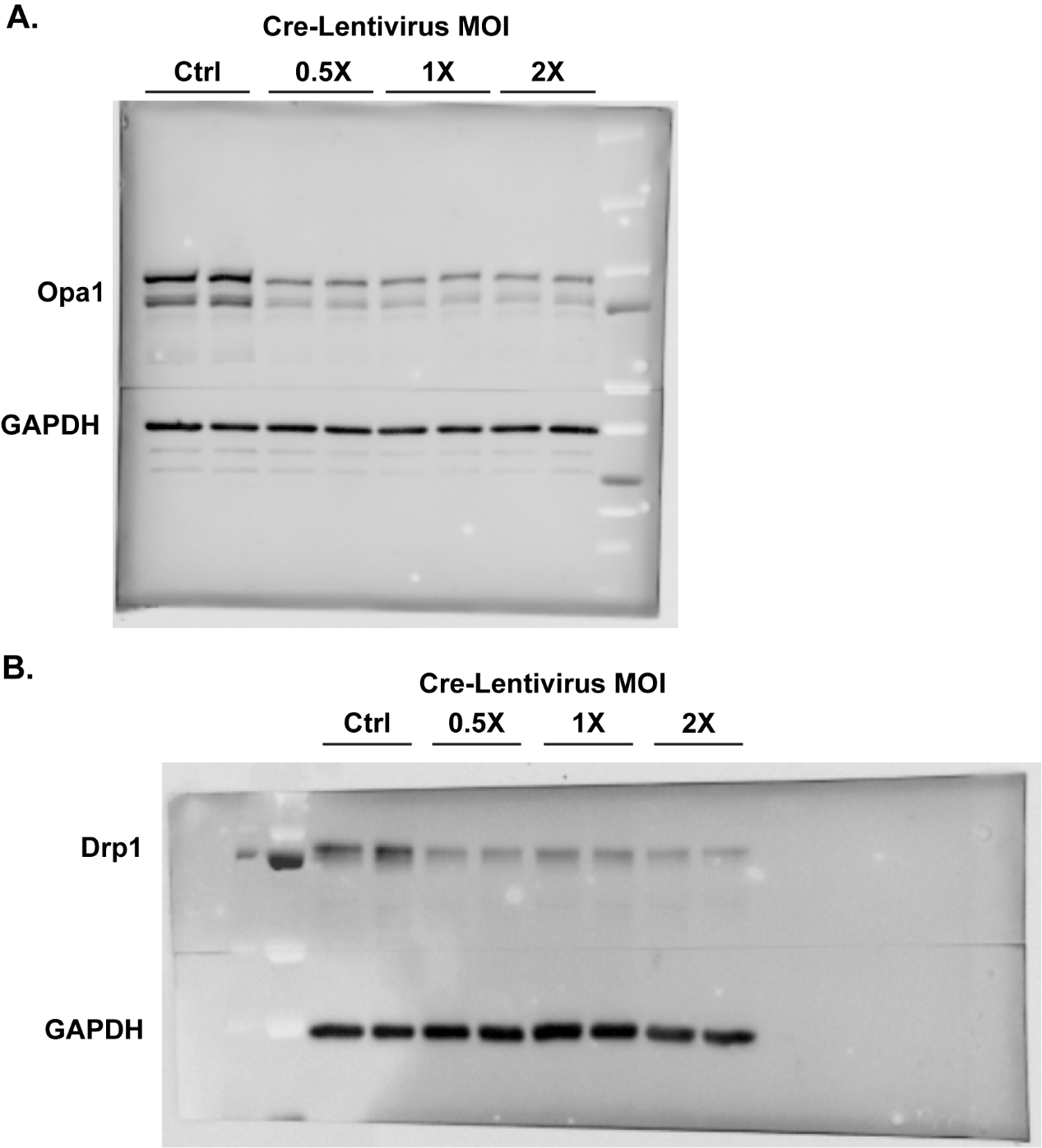
**

**Supplementary Figure 3. Full-length Western blots. (A)** Full-length Western blot displaying cKO of Opa1, corresponding to blot shown in Fig. 2C. **(B)** Full-length Western blot displaying cKO of Drp1, corresponding to blot shown in Fig. 3C.
